# Paradigm shift in utilization of blood and blood components during COVID pandemic among pregnant women in India

**DOI:** 10.6026/973206300222634

**Published:** 2026-04-30

**Authors:** Saritha Karre, Anitha Sunkara, Neelima Manda, Sreelakshmis Induri, Chandrakumar Shanmugam

**Affiliations:** 1 Department of Pathology, Government Medical College Quthbullapur, Malkajgiri, Medchal Telangana India

**Keywords:** COVID-19 pandemic, paradigm shift, pregnant woman, blood and blood components

## Abstract

The COVID-19 pandemic posed major challenges in obstetric care due to altered maternal physiology and coagulation abnormalities, increasing transfusion requirements in pregnant women. Blood and blood component transfusion remains essential for managing anemia, obstetric hemorrhage and sepsis. Therefore, it is of interest to evaluate the pattern and indications of blood and blood component utilization in COVID-positive pregnant women and compared them with the pre-COVID era. Packed red cells were the most frequently transfused component, followed by fresh frozen plasma and platelets during the pandemic. The observed change in utilization shows COVID- 19-related coagulopathy for adequate availability of blood components during pandemics.

## Background:

The emerging pandemic of the severe acute respiratory syndrome coronavirus 2 (SAR-CoV-2) is a global concern. In December 2019, Coronavirus-associated pneumonia was first reported in Wuhan city, China [1], since then it has been widespread in China and all over the world and infecting tens of millions of individuals [2]. This new corona virus disease entitled as Coronavirus disease 19 (COVID 19) by the World Health Organization (WHO) on February 11, 2020 and Taxonomy of viruses of international committee retitled the virus as severe acute respiratory syndrome coronavirus (SARS-CoV-2) [3]. Corona viruses are single stranded ribonucleic acid (RNA) virus, enveloped, non -segmented causing disease from common cold to severe fetal diseases. Owing to alterations in the immune response during pregnancy, these women are more susceptible to COVID 19 infection and its complications. The use of Blood and Blood components is crucial in obstetric practice; we evaluated its utilization in pregnant women during the COVID pandemic & compared it with Pre-COVID era. Normally, pregnant women need special care for the best pregnancy outcomes. But under the current situation, during the COVID-19 pandemic, these women require more care and attention, especially after the appearance of undesirable results on the mother and her baby by this virus [4] due to physiological changes of the immune and cardiopulmonary systems [5]. Pregnant women with corona virus disease in 2019 (COVID-19) represent a further challenge to clinicians, due to the risk of fetal toxicity, particularly during the first trimester of gestation. A quarter of cases of pneumonia in pregnancy require intensive care treatment with mechanical ventilation [6] and the most common complications that can occur are premature rupture of membranes (PROM), restriction of intrauterine growth (IUGR), premature labor, intrauterine and neonatal death can occur [7, 8]. Indeed, although the majority of them are asymptomatic or their SARS-CoV-2 disease has a mild to moderate course, in some cases this viral infection is accompanied by severe respiratory symptoms, common symptoms are fever and cough, whereas less common symptoms were myalgia, sore throat, diarrhea and shortness of breath [9]. Therefore, it is of interest to evaluate the utilisation patterns of Blood and Blood Components along with indications during COVID Pandemic as compared to Pre COVID Era in pregnant women.

## Materials and Methods:

A total of 678 COVID positive pregnant patients were selected for the present study, that was conducted for a period of 4years (March 2018- Feb 2022) in the usage of blood and blood components It is a retrospective study carried out in Department of Pathology in Gandhi Medical College & Hospital, Hyderabad, Telangana. 678 COVID19 positive (RT PCR +ve) pregnant women and RT PCR -ve along with ground glass opacities on HRCT chest were included. All pregnant women who are COVID negative, Recovered COVID positive pregnant women are excluded. A real time reverse transcriptase polymerase chain reaction (RT-PCR) assay is the gold standard for diagnosis. Chest Computed Tomography may aid in the diagnosis and can be used to assess the extent and follow-up of COVID-19. Pulmonary ultrasound has also been suggested for a quick diagnosis of pneumonia in pregnant women. Pregnancy is not a contraindication to blood component transfusion. Blood collection from the voluntary or replacements donors must be done in aseptic methods using a sterile closed system after the screening tests for Hepatitis, VDRL, HIV etc. To prevent activation of the coagulation system blood must be collected rapidly with a single venepuncture and with minimal trauma to the tissue. There should be frequent, gentle mixing of the blood with the anticoagulant. All the blood bags must be processed within 4 to 6 hours of phlebotomy. Blood collection bags constitute 350 ml or 450 ml capacity (single, double, triple/ quadruple) containing CPD/CPDA-1 as anticoagulant preservative solution. All the satellite bags must be accurately identified, numbered, labelled as coming from the original unit and must be accurately balanced before centrifugation. The whole blood which is a mixture of cells, colloids and crystalloids can be separated into different components namely packed red blood cell concentrate (PRBC), platelet concentrate, Fresh Frozen Plasma (FFP) and cryoprecipitate. The components are prepared by centrifugation of one unit of whole blood. Single component required can also be collected by apheresis procedure in blood donors. Different components need different storage conditions and temperature requirements for therapeutic efficacy. Equipments (Refrigerated centrifuges, Deep freezer, Blood bank refrigerator, Plasma extractor/ expressor, Hand sealer, roller and cutter, dielectric sealer, double pan balance, platelet rotator/agitator with incubator, thawing bath, cryobath, laminar flow cabinet) and consumables used for blood component preparation. Whole blood stored at 1-6 degree C and has a shelf life of 21 days. PRBC are stored in refrigerator /cold room at temp 2-6degree C and has a shelf life of 42 days if additive solution is added. Platelets can be stored at 22-24degree C in a platelet agitator and incubator for 72hrs or for 5 days depending on the quality of storage bags. FFP stored at -18degree C or colder and has a shelf life of 12 months. Cryoprecipitate is stored at -18degree C or colder and has a shelf life of 12 months. Blood and blood components issues for the transfusion are documented in the register named as labour room in the blood bank and the following data is collected for the present study. Cryoprecipitate is a plasma derived blood product for transfusion that contains fibrinogen, factor XIII, factor VIII, von Willebrand factor and fibronectin. Fresh frozen plasma is a plasma derived blood product for transfusion that contains fibrinogen, plasma proteins, electrolytes, physiological anticoagulants (protein C, protein S, antithrombin). Random Donor Platelets are called as Pooled platelets, obtained from whole blood in routine blood donations. Convalescent Blood Plasma (CBP) transfusion depends mainly on separating blood plasma from individuals whom have successfully overcome infection, using the specific antibodies-evolved by their immune system- or combat the same invader in other patients, with main concern to eradicate the pathogen. In the Current situation Convalescent blood plasma is not in use.

## Results:

Out of 678 COVID positive pregnant women who were selected for the present study, that was conducted for a period of 4years (March 2018-Feb 2022) in the usage of blood and blood components in COVID positive pregnant women (March 2020-Feb 2022) and are compared with Pre COVID Cases (March 2018-Feb 2020). The transfusion of Blood (60.32%) and Blood components (39.68%) of COVID positive pregnant women and the transfusion of blood (67.2%) and blood components (32.8%) of Pre COVID pregnant women correlation observed are given in (Figure 1 & Table 1 - see PDF) with Chi square value= 12.7, Df=1 (Degree of freedom), P Value= 0.00017. Blood transfusion in Pre COVID pregnant patients is more when compared to COVID positive pregnant patients. Blood components transfusion in COVID positive pregnant patients is more when compared to Pre COVID pregnant patients. The transfusion of blood and blood components in COVID positive pregnant women observed are given in Table 2 (see PDF), The majority of patients transfused Packed cells (60.11%) followed by Fresh Frozen Plasma (22.82%),Platelets (16.43%), Cryo precipitate (0.50%) & Whole Blood (0.14%).The transfusion of blood and blood components in Pre COVID pregnant women observed are given in (Table 2 - see PDF), The majority of patients transfused Packed cells (40.72%) followed by Whole Blood (26.48%),Fresh Frozen Plasma (18.03%),Platelets (14.12%), Cryoprecipitate (0.65%). The transfusion of blood (60.32%) and blood components (39.68%) of COVID positive pregnant women and the transfusion of blood (67.2%) and blood components (32.8%) of Pre COVID pregnant women correlation observed are given in (Table 2 - see PDF) with Chi square value= 12.7, Df=1 (Degree of freedom), P Value= 0.00017. Blood transfusion in Pre COVID pregnant patients is more when compared to COVID positive pregnant patients. Blood components transfusion in COVID positive pregnant patients is more when compared to Pre COVID pregnant patients. In the present study out of 678 COVID positive pregnant women who were selected were studied with respect to age are given in (Table 3 - see PDF). Patient age ranged from 21-30 years, majority of patients (97.89%) were transfused with packed cells, fresh frozen plasma, platelets and cryoprecipitate. Most common clinical presentation was fever and cough, whereas less common symptoms were myalgia, malaise, sore throat, diarrhoea and shortness of breath. The transfusion of blood and blood components in 1st, 2nd & 3rd trimester in COVID positive pregnant women and Pre COVID pregnant women correlation observed are given in (Figure 2 & Table 4 - see PDF) with Chi square value= 0.0016, Df=2 (Degree of freedom), P Value= 0.9992. Blood transfusion in Pre COVID pregnant patients is more when compared to COVID positive pregnant patients in 3rd trimester of pregnancy. Indications of Transfusion of Whole Blood in COVID positive and Pre COVID Pregnant Women in (Table 5 - see PDF). Main Indications of Transfusion of Whole Blood in COVID cases is Anaemia and in pre-COVID cases in addition to Anaemia, Atonic PPH (Postpartum Haemorrhage), Abruption, retained placenta and AFLP (AFLP-Acute Fatty Liver of Pregnancy) are included. Indications of Transfusion of Packed RBCs in COVID positive and Pre COVID Pregnant Women in (Table 6 - see PDF), commonest indication was Anaemia in COVID positive & Pre COVID cases followed by sepsis in COVID cases & Atopic PPH in Pre COVID cases during Pre COVID era. (Miscellaneous- Ectopic Pregnancy; Ruptured Uterus; Post-Operative Sepsis; Fibroid Complicating Pregnancy) Indications of Transfusion of Platelets in COVID Positive and Pre COVID Pregnant Women in (Table 7 - see PDF), commonest indication was Thrombocytopenia in COVID positive & Pre COVID cases followed by HELLP in COVID cases & in Pre COVID cases during Pre COVID era. Indications of Transfusion of Fresh Frozen Plasma in COVID Positive and Pre COVID Pregnant Women in (Table 8 - see PDF), commonest indication was HELLP (Haemolysis, Elevated Liver enzymes, Low Platelet count) in COVID positive & Pre COVID cases followed by DIC in COVID cases & Atonic PPH in Pre COVID cases during Pre COVID era. Indications of Transfusion of Cryoprecipitate in COVID Positive and Pre COVID Pregnant Women in (Table 9 - see PDF), commonest indication was HELLP, DIC, Atonic PPH in COVID positive & in Pre COVID cases HELLP followed by DIC. Chi square value= 12.7, DF=1 (Degree of freedom), P Value= 0.00017. Blood transfusion in Pre COVID pregnant patients is more when compared to COVID positive pregnant patients. Blood components transfusion in COVID positive pregnant patients is more when compared to COVID Pre COVID pregnant patients. Among the Blood components Packed RBC'S were utilized more frequently in both the periods (Table 10 - see PDF). Among the Blood components least utilized was Cryoprecipitate. Chi square value= 0.0016, DF=2 (Degree of freedom), P Value= 0.9992. Blood transfusion of blood components in COVID positive pregnant women is more when compared to pre-COVID pregnant women in 3rd trimester of pregnancy.

## Discussion:

Pregnant women usually require special care for the best pregnancy outcomes. However, in the current situation, these women are affected during the COVID-19 pandemic, especially after the undesired consequences of this virus [10] in mothers and babies due to physiological changes in the immune and cardiopulmonary systems. It requires more attention [11]. The transfusion of blood and blood components in COVID positive pregnant women, majority of patients transfused packed cells (60.11%) followed by fresh frozen plasma (22.82%), platelets (16.43%), Cryo precipitate (0.50%) & whole blood (0.14%). The majority of COVID-19- positive pregnant women in this study were asymptomatic or had only mild symptoms, but those with underlying disorders should be given special attention because they have a larger chance of developing severe disease than the general population. SARS has been linked to a high rate of miscarriage in pregnant women. Due to the lack of evidence on COVID-19 infection in the first trimester, an increased risk of miscarriage in women with COVID-19 cannot be ruled out at this time. In a living systematic review by Allotey and colleagues [12] on maternal and perinatal outcomes of COVID-19 and pregnancy, increased maternal age (>35 years), obesity, hypertension and pre-existing diabetes were associated with a severe COVID-19. To date, there is limited data about the effectiveness of therapies used for COVID-19, particularly in pregnant women. Evidences show that convalescent plasma from patients who have recovered from viral infections was used as a treatment without the occurrence of severe adverse events in general population [13]. First trimester Li *et al.* reported that four of seven pregnant women with SARS presenting in the first trimester had spontaneous miscarriages and two had elective terminations. The only new-born survivor was delivered at term and no anomalies were reported [14]. Blood transfusion in COVID negative pregnant patients is more when compared to COVID positive pregnant patients in 3rd trimester of pregnancy. Kumar *et al.* in their similar study found that, the leading reason for admission in both the COVID era and pre-COVID era transfused patients was shortness of breath (53.7% and 36% P = 0.001), followed by gastrointestinal bleeding (25.9% and 21% P = 0.001). There was a higher percentage of RBC transfusions in the intensive care unit in the COVID-era group than in the pre-COVID era group (38.9% vs 22%, P = 0.008). The restrictive transfusion criteria were met in 62% vs 79% in the COVID and pre-COVID eras, respectively (P = 0.008). Thus, they conclude that, the COVID-era group received RBC transfusions with less stringent adherence to restrictive blood transfusion practices in comparison to pre- COVID era group [15]. Another study done by Solanki *et al.* found that the blood collection has been reduced to 46.6%, demand for blood has declined to 54.5% and the blood supply has also been reduced to 67.0% during the COVID-19 pandemic. The demand for blood in females was relatively higher than in male patients. Maximum demands of packed blood cells were recorded for O-positive blood group patients in 30-39 years of age with severe anaemia, followed by cancer and trauma. The demand for blood was more as compared to blood collection and supply. This gap between demand and supply showed a negative impact of the COVID-19 pandemic on BTS. Therefore, they come to conclude that, the COVID-19 pandemic had a negative impact on donor attendance and participation. Thus, adversely affects the BTSs in sustaining the equilibrium between blood demand and blood supply [16]. Another study had showed that, at the Department of Anesthesiology and Intensive Therapy, the median weekly ratio of transfused patients fell from 50% (pre-pandemic) to 9.76% (third wave of pandemic). COVID-19 diagnosis was associated with lower odds of receiving transfusion (OR: 0.23) and with a lower incidence rate ratio of transfused red blood cells (IRR: 0.22). At the Department of Surgery, the median weekly ratio of transfused patients was consistently low and stable (9-10%) throughout the study period. The number of patients remained relatively stable at the Division of Hematology during the study period, expressing a higher odds of receiving transfusion during the second (OR: 2.63) and fourth (OR: 1.52) pandemic waves. Thus, they conclude that, the pandemic's impact on transfusion practice, driven by indirect various consequences of patient redirection and protocol modifications, was most expressed at the Department of Anesthesiology and Intensive Therapy [17].

## Conclusion:

We observed a paradigm shift in the pattern and frequency of Utilization of Blood and Blood Components during COVID pandemic verses Pre COVID era. The indication during COVID Pandemic points to coagulation abnormalities due to COVID 19 disease. Hence Blood components especially Platelets, FFP's & cryoprecipitate have to be made available in good numbers during ongoing COVID 19 pandemic for better obstetric outcome which could be a life saviour.
